# Histopathological study of JNK in venous wall of patients with chronic venous insufficiency related to osteogenesis process

**DOI:** 10.7150/ijms.54052

**Published:** 2021-03-03

**Authors:** Miguel A Ortega, Ángel Asúnsolo, Leonel Pekarek, Miguel A Alvarez-Mon, Arnaud Delforge, Miguel A Sáez, Santiago Coca, Felipe Sainz, Melchor Álvarez- Mon, Julia Buján, Natalio García-Honduvilla

**Affiliations:** 1 Department of Medicine and Medical Specialities, Faculty of Medicine and Health Sciences, University of Alcalá, Alcalá de Henares, Madrid, Spain.; 2 Ramón y Cajal Institute of Healthcare Research (IRYCIS), Madrid, Spain.; 3 Cancer Registry and Pathology Department, Hospital Universitario Principe de Asturias, Alcalá de Henares, Madrid, Spain.; 4 Department of Surgery, Medical and Social Sciences, Faculty of Medicine and Health Sciences, University of Alcalá, Alcalá de Henares, Madrid, Spain.; 5 UFR of pharmacy, University of Clermont Auvergne, Clermont-Ferrand, France.; 6 Pathological Anatomy Service, Central University Hospital of Defence-UAH Madrid, Spain.; 7 Angiology and Vascular Surgery Service, Central University Hospital of Defence-UAH Madrid, Spain.; 8 Immune System Diseases-Rheumatology, Oncology Service and Internal Medicine, University Hospital Príncipe de Asturias, Alcalá de Henares, Madrid, Spain.

**Keywords:** chronic venous insufficiency, venous reflux, JNK, osteogenesis, ageing

## Abstract

Chronic venous insufficiency (CVI) is one of the most common vascular pathologies worldwide. One of the risk factors for the development of CVI is aging, which is why it is related to senile changes. The main trigger of the changes that occur in the venous walls in CVI is blood flow reflux, which produces increased hydrostatic pressure, leading to valve incompetence. The cellular response is one of the fundamental processes in vascular diseases, causing the activation of cell signalling pathways such as c-Jun N-terminal kinase (JNK). Metabolic changes and calcifications occur in vascular pathology as a result of pathophysiological processes. The aim of this study was to determine the expression of JNK in venous disease and its relationship with the role played by the molecules involved in the osteogenic processes in venous tissue calcification. This was a cross-sectional study that analyzed the greater saphenous vein wall in 110 patients with (R) and without venous reflux (NR), classified according to age. Histopathological techniques were used and protein expression was analysed using immunohistochemistry techniques for JNK and markers of osteogenesis (RUNX2, osteocalcin (OCN), osteopontin (OPN)). Significantly increased JNK, RUNX2, OCN, OPN and pigment epithelium-derived factor (PEDF) protein expression and the presence of osseous metaplasia and amorphous calcification were observed in younger patients (<50 years) with venous reflux. This study shows for the first time the existence of an osteogenesis process related to the expression of JNK in the venous wall.

## Introduction

Chronic venous insufficiency (CVI) is a term that describes functional anomalies of the venous system 1,2. CVI is a multifactorial disease that can lead to valve incompetence and venous hypertension, causing venous reflux 3,4. Risk factors include family history, sedentary lifestyle, age, female sex, pregnancy and obesity 5-7. Varicose veins are a common manifestation of CVI. Both entities, varicose veins and CVI, can be summarized under the term chronic venous disorders, which includes the full spectrum of morphological and functional anomalies of the venous system 8. It is estimated that more than 30 million adults in the United States have some manifestation of this disease 9. Different epidemiological studies conducted worldwide show that CVI is a chronic pathology with large variability in incidence and prevalence 8,10. The prevalence of CVI is increasing, causing disability and creating an important socioeconomic problem 11.

The main trigger for the changes that occur in the venous wall in CVI is blood flow reflux, which produces increased hydrostatic pressure 3,12. Venous reflux creates a pathological situation where a blood stasis occurs that alters the homeostasis of the venous wall as an adaptive mechanism to this hyperpressure situation. This leads to a greater amount of blood in the venous segment preceding the valve failure, producing venous hypertension 13. Events that modify valve structure, such as stretching, splitting, tears, thinning and adhesion, trigger valve incompetence 14,15. This can lead to weakening of the structural integrity of the venous wall and its remodelling, in addition to other changes at different levels that give rise to the clinical manifestations of CVI 16-20.

The activation of cell signalling pathways is one of the most important mechanisms for understanding how tissues respond to different events that alter cellular homeostasis 21. Numerous studies have demonstrated the importance of the c-Jun N-terminal kinase (JNK) pathway in these processes, as it responds to and regulates the pathophysiological changes that occur 22. The importance of JNK dysregulation lies in the impact it has on gene expression, cell viability and the extracellular matrix, affecting the effectiveness of the tissues of the involved organ 23-25. JNK has a great importance in diseases of the vascular system, since the hemodynamic changes that occur in these diseases cause its expression to be altered with consequences in the tissues 26.

Among these events, calcification with the hydroxyapatite (HA) presence in venous wall may play a fundamental role in the JNK activation. This pathway has been related to expression of osteogenic genes induced by HA and the osteogenic differentiation of smooth muscle cells. The calcification processes, as it plays a determinant role in in calcium deposition in smooth muscle cells 27,28.

Biomineralization of tissues has been described as a common process in tissues involved in chronic vascular diseases 29,30. Increased clinical and experimental evidence suggests that inflammation accelerates the progression of calcification, as molecules in common with bone metabolism are activated 31-33. Recent studies have demonstrated that RUNX2, osteopontin (OPN) and molecules involved in similar metabolic pathways play central roles in the calcification of atherosclerotic lesions and in calcification in heart disease 30,33-35. Abnormalities in the equilibrium of these proteins can cause disturbances in vascular and valvular calcification 36,37. In this line, it has been noted that the activity of pigment epithelium-derived factor (PEDF) and the presence of HA becoming involved in the activation of the JNK pathway as a response of osteogenic differentiation of vascular cells 38-42.

Therefore, the aim of this study was to determine JNK expression and its relationship with the mineralization of venous tissue in patients with and without valve incompetence (clinically diagnosed with venous reflux) according to age, observing the presence of specific markers of bone metabolism, such as RUNX2, osteocalcin (OCN) and OPN, as well as of PEDF.

## Methods

### Experimental design

This was a cross-sectional study in patients with CVI (n=110) scheduled to undergo saphenectomy stratified by age younger than 50 years or 50 years or older. ***Inclusion criteria*** were men or women with CVI with and without reflux in the great saphenous vein, a body mass index (BMI) ≤25 kg/m2, signed informed consent and a commitment to pre- and postoperative follow-up and tissue sample donation. Patients were excluded (***Exclusion criteria)*** if they had vein malformations or arterial insufficiency, lacked medical records, had a pathology affecting the cardiovascular system (infectious disease, diabetes, dyslipidemia, hypertension) excluding chronic venous insufficiency. Patients with toxic habits were excluded: smoking, alcohol or drug abuse and if they thought they would not be able to attend all the follow-up sessions scheduled. 110 patients were recruited for our study. These patients were classified as venous reflux (R) n=81(50.09±15.91 years) or no venous reflux (NR) n=29 (51.51±14.04 years). These groups were then divided by age into subgroups of patients with reflux younger than 50 years (R<50) n=32 (35.09±7.31 years) or 50 years or older (R≥50) n=49 (59.98±11.81 years). Furthermore, patients without reflux younger than 50 years (NR<50) n=13 (38.53±6.21 years) or 50 years or older (NR≥50) n=16 (62.06±8.54 years). There were no significant differences in the hemogram or in the general biochemistry (data not shown).

The study protocol was conducted according to the basic principles of healthcare ethics and according to Good Clinical Practice guidelines, the principles of the last Declaration of Helsinki (2013) and the Oviedo Convention (1997). Patients were duly informed and asked to sign an informed consent form. The Clinical Research Ethics Committee of Central University Hospital of Defense-UAH approved the study protocol (37/17).

Each candidate patient was examined using an Eco-Doppler color M Turbo (Sonosite, Bothell, WA, USA ) 7.5 Mz probe. The lower limbs were examined with the patient standing with the leg under examination rotated outwards. The exam comprised the greater saphenous from the groin to the ankle and the femoral vein. Examination of the small saphenous and popliteal veins was also performed with the patient standing with the back towards the examiner and the leg non-weight bearing. In this study, the Valsalva manoeuvre (it is any attempt to exhale air with the glottis closed or with the mouth and nose closed, increasing the pressure inside the thorax) was performed whereby proximal circulatory arrest allows for examination of the venous insufficiency proximal to the detection point and leakage points are identified (assessing the lack of reflux at the femoroiliac and saphenofemoral junctions). To assess trunk venous flow direction, the distal compression and decompression manoeuvre was performed. Reflux was defined as a duration of venous reflux (DVR) longer than 0.5 s. NR patients had a compressive syndrome (Nutcracker syndrome). Once the saphenous vein was harvested, its entire length was cut into fragments and these were introduced into two sterile tubes: one containing minimal essential medium (MEM) supplemented with an antibiotic and antimycotic at 1% (both from Thermo Fisher Scientific, Walthan, MA, USA).

### Structural and ultrastructural studies

Tissue samples were processed in a sterile environment (class II laminar flow cabinet Telstar AV 30/70 Müller 220 V 50 MHz, Grupo Telstar SA, Terrassa, Spain). Samples were kept in 1 mL of RNAlater® at -80 °C until processing for gene expression analysis. Samples in MEM were used in the histological study of the vein tissue. Samples were washed/hydrated several times in MEM without antibiotic to remove blood cells and cut into pieces for maintenance in two different fixing solutions: F13 (60% ethanol, 20% methanol, 7% polyethylene glycol, 13% distilled H_2_O) or 3% glutaraldehyde. Once the samples were fixed, they were dehydrated and embedded in paraffin. The paraffin blocks were cut with a rotation HM 350 S microtome (Thermo Fisher Scientific Walthan, MA, USA) to obtain 5 µm thick sections on glass slides coated with a 10% poly-lysine solution. Once dry, the sections were deparaffinated for 30 min in xylol (PanReac AppliChem, Barcelona, Spain) and then rehydrated by passes through a decreasing alcohol series. Next, the sections were subjected to different staining (Von Kossa and PTAH) and immunohistological techniques 9.

### Von Kossa staining for calcium deposits

Using this stain, calcium deposits appear brown-black on a red background. The protocol followed was: 1. Incubate slide in sodium nitrate solution for 30 min, 2. Wash in sodium thiosulphate solution (5%) for 15 min, 3. Rinse in running water, 4. Incubate slide in Fast Red for 1 min, 5. Dehydrate in 96% alcohol for 3 min, 6. Dehydrate in 100 % alcohol for 5 min, 7. Clear sections in xylol for 10 min, 8. Mount slides with Cytoseal™ 43.

### Phosphotungstic acid haematoxylin (PTAH) staining for fibrin

Using this stain, fibrin appears dark blue while nuclei, cytoplasms, red blood cells and fibrils appear pale blue or purple. Collagen takes on a red tone. The protocol 44 followed was: 1. Incubate slide in 50% Lugol's iodine until a yellow-brown colour is acquired, 2. Eliminate excess iodine and wash in 0.5% potassium thiosulphate solution, 3. Wash in distilled water for 5 min, 4. Incubate slide in 5% sodium thiosulphate for 3 min, 5. Rinse in distilled water, 6. Incubate in an oxalate solution for 1 min, 7. Rinse in distilled water, 8. Stain with PTAH for 2 h at 60 °C, 9. Dehydrate: 2 × 1 min passes in 96% alcohol and 2 × 1 min passes in 100% alcohol, 10. Clear sections in xylol for 10 min. 11. Slides were mounted with Cytoseal™.

For ***transmission electron microscopy***(TEM), small fragments of vein tissue were introduced into 3% glutaraldehyde for 1-2 h. Next, the fixed samples were washed in Millonig buffer for at least 2 h. Embedding was done over three consecutive days. Semi-thin sections of 1 μm thickness were cut with an Ultracut Reichert-Jung (Reichert Technologies, Depew, NY, USA) ultramicrotome and then stained with Toluidine blue and visualized under a light microscope. Once the area of interest was identified, the blocks were carved with a shaper (Creighert-TM 60), and ultra-thin sections of 60 nm prepared. The sections were collected on copper grids coated with a Formvar resin membrane and treated with lead citrate for 4 min. Once washed and dried, the sections were visualized with a tabletop TM microscope (TM-100).

### Immunohistochemistry

Antigen-antibody reactions were detected by the ABC method 45,46 (avidin-biotin complex) using peroxidase or alkaline phosphatase as a chromogen according to the following protocol. 1. The samples were washed with 1× PBS 3 times (5 minutes each). 2. Non-specific binding sites were blocked with 3% BSA (bovine serum albumin) in PBS for 30 minutes at room temperature. 3. The samples were incubated with primary antibody (Table 1A) diluted in 3% BSA and PBS overnight at 4 °C. 4. The samples were washed with PBS 3 times (5 minutes each). 5. The samples were incubated with biotin-conjugated secondary antibody (Table 1B) diluted in PBS for 1 hour and 30 minutes at room temperature. 6. The samples were washed with PBS 3 times (5 minutes each). 7. The samples were incubated with ExtrAvidin®-Peroxidase (Sigma-Aldrich, St. Louis, MO, USA), diluted 1/200 in PBS, for 1 hour at room temperature.; for ExtrAvidin®-Alkaline Phosphatase (Sigma-Aldrich), the samples were incubated for 60 minutes at room temperature (dilution 1/200 in PBS). 8. The samples were washed in PBS 3 times (5 minutes each). 8A. For development, the samples were incubated with the chromogenic substrate diaminobenzidine (DAB, SK-4100) (Vector, Burlingame, CA, USA); the chromogenic substrate was prepared immediately before development: 5 mL of distilled water, 2 drops of buffer, 4 drops of DAB, and 2 drops of hydrogen peroxide. This technique results in brown staining. 8B. For the immunodetection of RUNX2 and PEDF, the samples were developed with alkaline chromogenic substrate for 15 minutes (appearance of staining was controlled under the microscope); the chromogenic substrate was prepared immediately before development: 10 mL of PBS, 10 mg of α-naphthol AS-BI phosphate, 10 mg of Fast red, and 100 μl of 0.1 M levamisole. 9. The samples were washed 3 times (5 minutes each) with distilled water to stop the development reaction. 10. Nuclei were stained with Carazzi's haematoxylin for 5-15 minutes. 11. The samples were washed in running water for 10 minutes. 12. The samples were mounting using plasdone aqueous medium. For all immunohistochemical assays, sections of the same tissue were used as a negative control, in which incubation with primary antibody was replaced with incubation in blocking solution.

### Statistical analysis and interpretation of the results

For statistical analysis, GraphPad Prism® 5.1 was used. The Mann-Whitney U test was applied for quantitative variables, and Fisher's exact test was used when applicable. The data are expressed as the median and interquartile range (IQR). The error bars in the figures correspond to the IQR. Significance was established at p < 0.05 (*), p < 0.005 (**), p < 0.001 (***). For each of the patients in the established groups, 5 sections and 10 fields per section were examined, by random selection. Samples were described as positive when the mean labelled area in the analysed sample was greater than or equal to 5% of the total, according to anatomopathological protocols 47,48. Evaluated the expression intensity for the immunohistochemical stain by scoring from 0 (negative) to 3. Thus, participants' histological specimens were classified as: 0-1, minimum staining (0-25%); 2, moderate staining (25-65%); 3, strong staining (65-100%), this procedure is similar to immunoreactive score (ISR score) 2, 49. The observation and quantification was carried out independently by 2 of the authors (M.A.O. and S.C.). The infiltrated cells were counted under a microscope (1000X) in 10 random areas (0.5 mm^2^ per patient) according to the method described by Ortega et al. 19. The samples were examined under a Zeiss Axiophot optical microscope (Carl Zeiss, Germany) equipped with an AxioCam HRc digital camera (Carl Zeiss, Germany).

## Results

### JNK activation in the venous wall of patients with CVI

**Figure 1:**
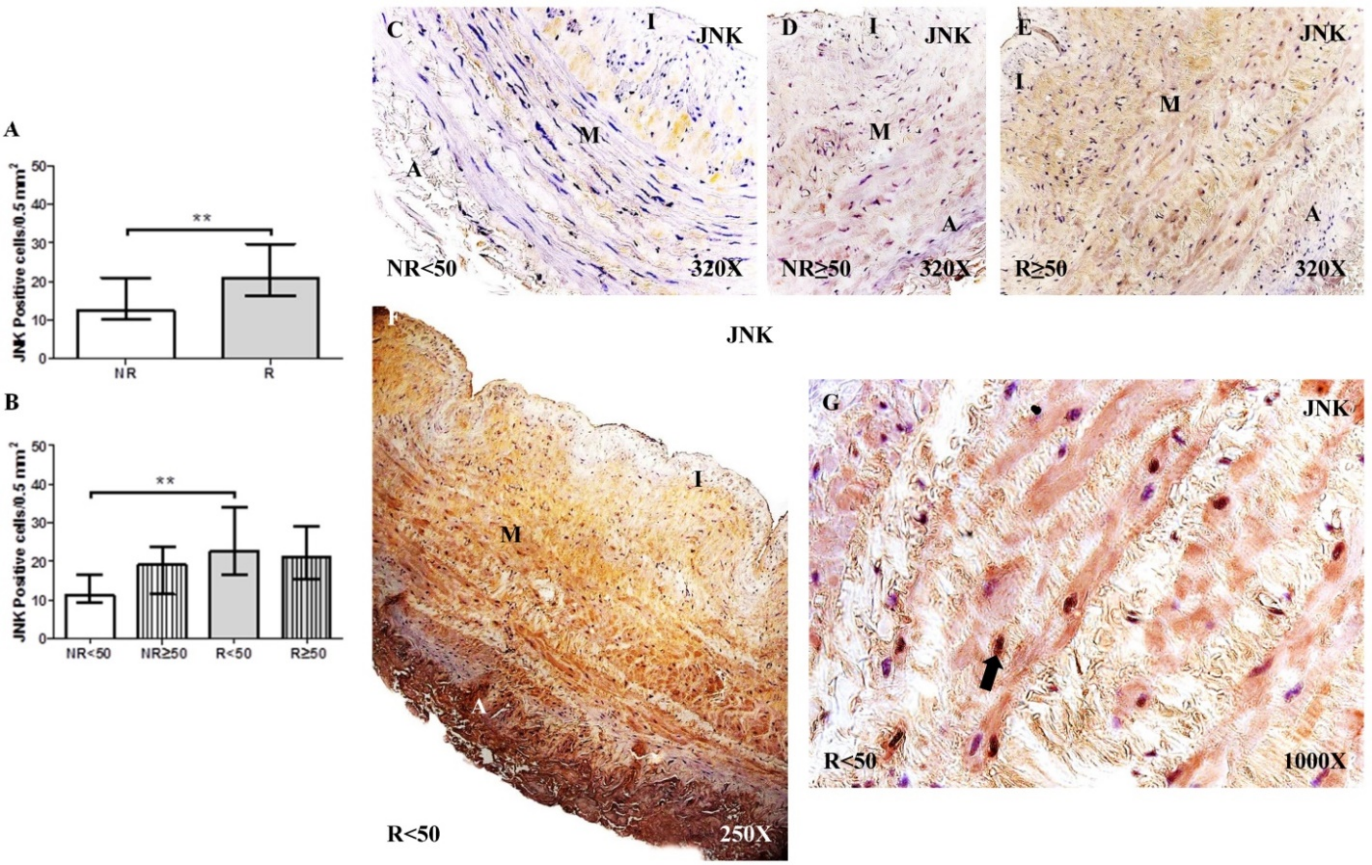
** A-B.** Quantification of the number of positive cells for the immunodetection of c-Jun N-terminal Kinase (JNK) by Immunohistochemistry technics per field in the different study groups. **C-G.** Representative images of JNK protein expression in the middle layer (arrow = immunoprecipitation). NR<50=patients without reflux younger than 50 years, NR≥50= patients without reflux with 50 years or older, R<50: patients with reflux younger than 50 years, R≥50: patients with reflux with 50 years or older. I, intimal layer; M, middle layer; A, adventitial layer. ** p <0.005.

The study of JNK expression in the saphenous vein wall revealed a significant increase in JNK expression in patients with valve incompetence clinically diagnosed as venous reflux (R) compared to patients without venous reflux (NR), ** p = 0.0035 (NR = 12.50 [7.00-31.00], R = 21.00 [14.00-41.00], Figure 1A). Regarding patient age, patients with R < 50 years old had the highest number of cells with positive JNK expression. Significance difference in JNK expression was observed between the NR < 50 years old group and R < 50 years old group, **p = 0.0044 (NR < 50 years old = 11.00 [7.00-21.00], NR ≥ 50 years old = 19.00 [9.00-31.00], R < 50 years old = 22.50 [14.00-41.00], R ≥ 50 years old = 21.00 [14.00-32.00], Figure 1B). The JNK protein expression was predominantly localized in the smooth muscle cells of the layer media (Figure 1C-G).

### Elevation of osteogenesis markers in the venous wall of patients with CVI

#### Expression of RUNX2

**Figure 2:**
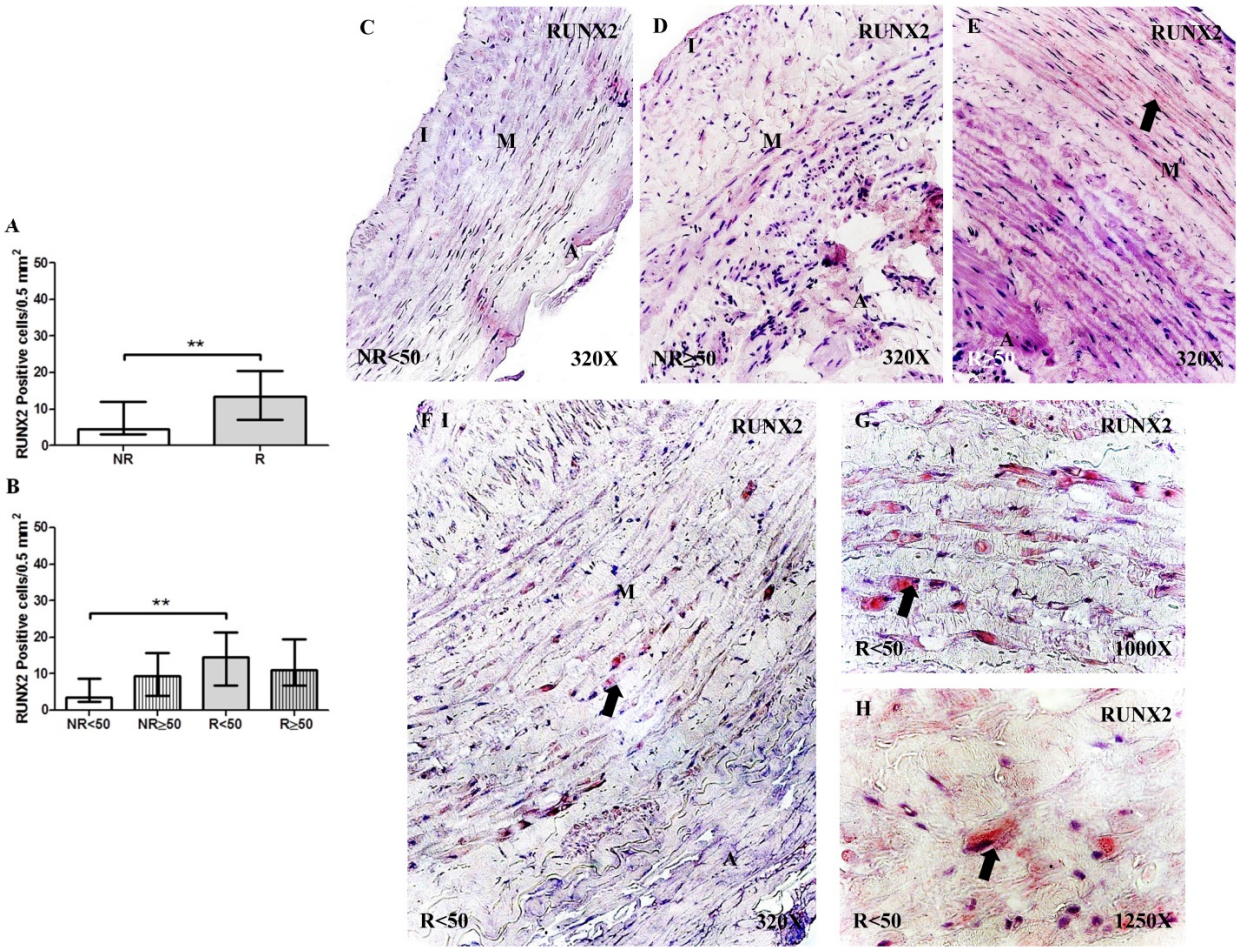
** A-B.** Quantification of the number of positive cells for immunodetection of RUNX2 by Immunohistochemistry technics per field in the different study groups**. C-H.** Representative images of the protein expression of RUNX2 in the middle layer (arrow: immunoprecipitation). NR<50: patients without reflux younger than 50 years, NR≥50: patients without reflux with 50 years or older, R<50: patients with reflux younger than 50 years, R≥50: patients with reflux with 50 years or older. I, intimal layer; M, middle layer; A, adventitial layer. ** p <0.005.

The determination of RUNX2 protein expression by immunohistochemical techniques revealed a significant increase in the number of positive cells in patients with R than in patients with NR, **p = 0.0041 (NR = 4.50 [24.00-21.00], R = 13.50 [5.00-25.00], Figure 2A). Regarding patient age, patients with R < 50 years old had the highest RUNX2 expression levels, differing significantly from that of patients with NR < 50 years old, **p = 0.0033 (NR < 50 = 3.50 [2.00-12.00], NR ≥ 50 = 9.50 [3.00-21.00], R < 50 = 14.50 [5.00-25.00], R ≥ 50= 11.00 [5.00-23.00], Figure 2B). RUNX2 expression was visualized in the cytoplasm of the cells of the layer media (Figure 2C-H).

#### Expression of osteocalcin (OCN)

**Figure 3:**
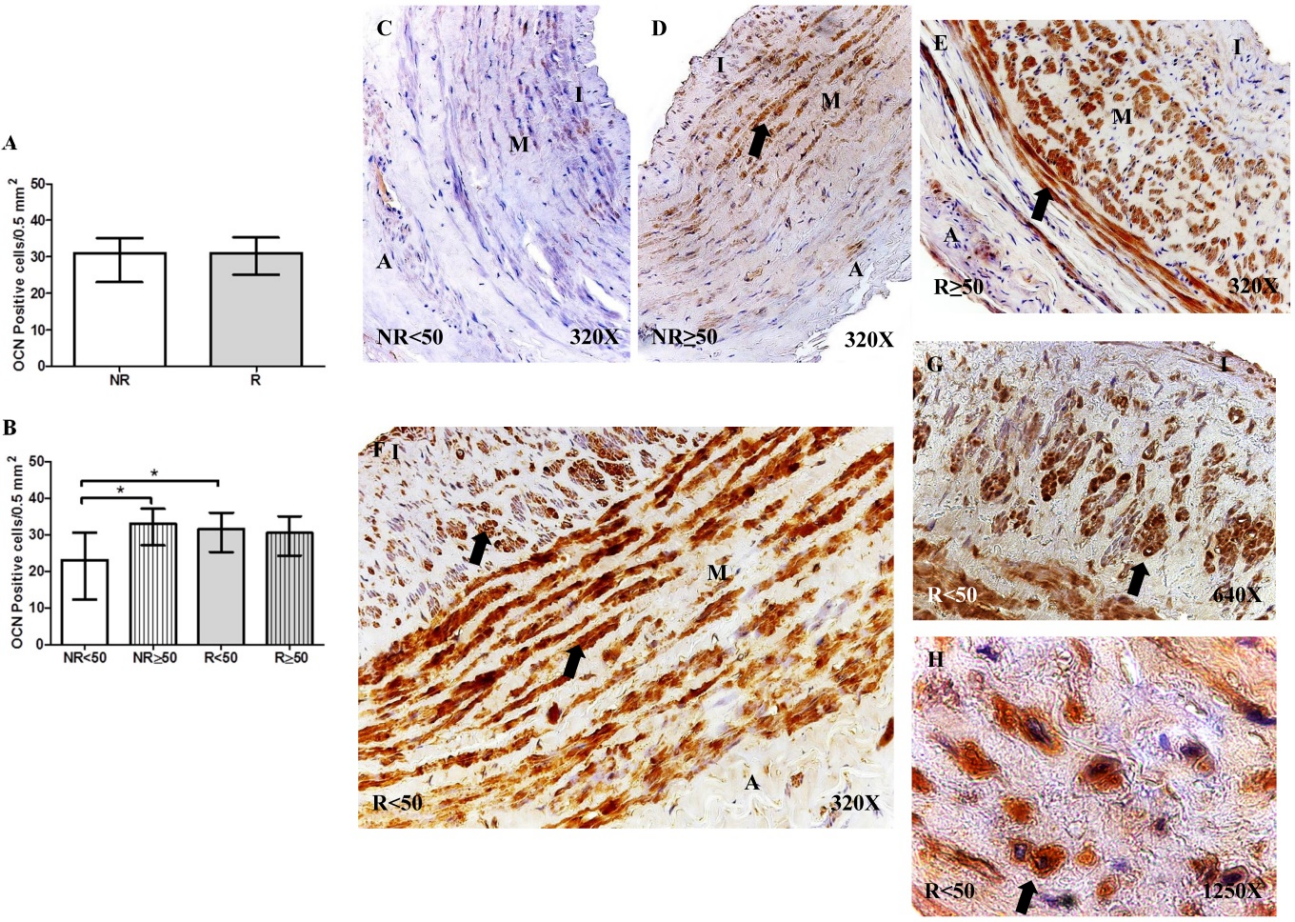
** A-B.** Quantification of the number of positive cells for the immunodetection of Osteocalcin (OCN) by Immunohistochemistry technics per field in the different study groups. **C-H.** Representative images of the protein expression of OCN in the middle layer (arrow: immunoprecipitation). NR<50: patients without reflux younger than 50 years, NR≥50: patients without reflux with 50 years or older, R<50: patients with reflux younger than 50 years, R≥50: patients with reflux with 50 years or older. I, intimal layer; M, middle layer; A, adventitial layer. * p <0.05.

OCN protein expression was not significantly different regarding the number of positive cells per field in the different study groups (NR = 30.50 [11.00-39.50], R = 31.00 [21.00-40.00], Figure 3A). However, OCN protein expression was significantly increased in patients with NR ≥ 50 years old compared to that of patients with NR < 50 years old, *p = 0.0186. Furthermore, a significant increase in OCN expression was observed in patients with R < 50 years old compared to that in patients with NR < 50 years old, *p = 0.0049 (NR < 50= 23.00 [11.00-36.00], NR ≥ 50 = 33.00 [23.00-39.50], R < 50= 31.50 [21.00-40.00], R ≥ 50= 30.50 [21.00-37.00], Figure 3B). OCN expression was visualized in the smooth muscle cells of the layer media and in the myointimal cells of the venous wall (Figure 3C-H).

#### Expression of osteopontin (OPN)

**Figure 4:**
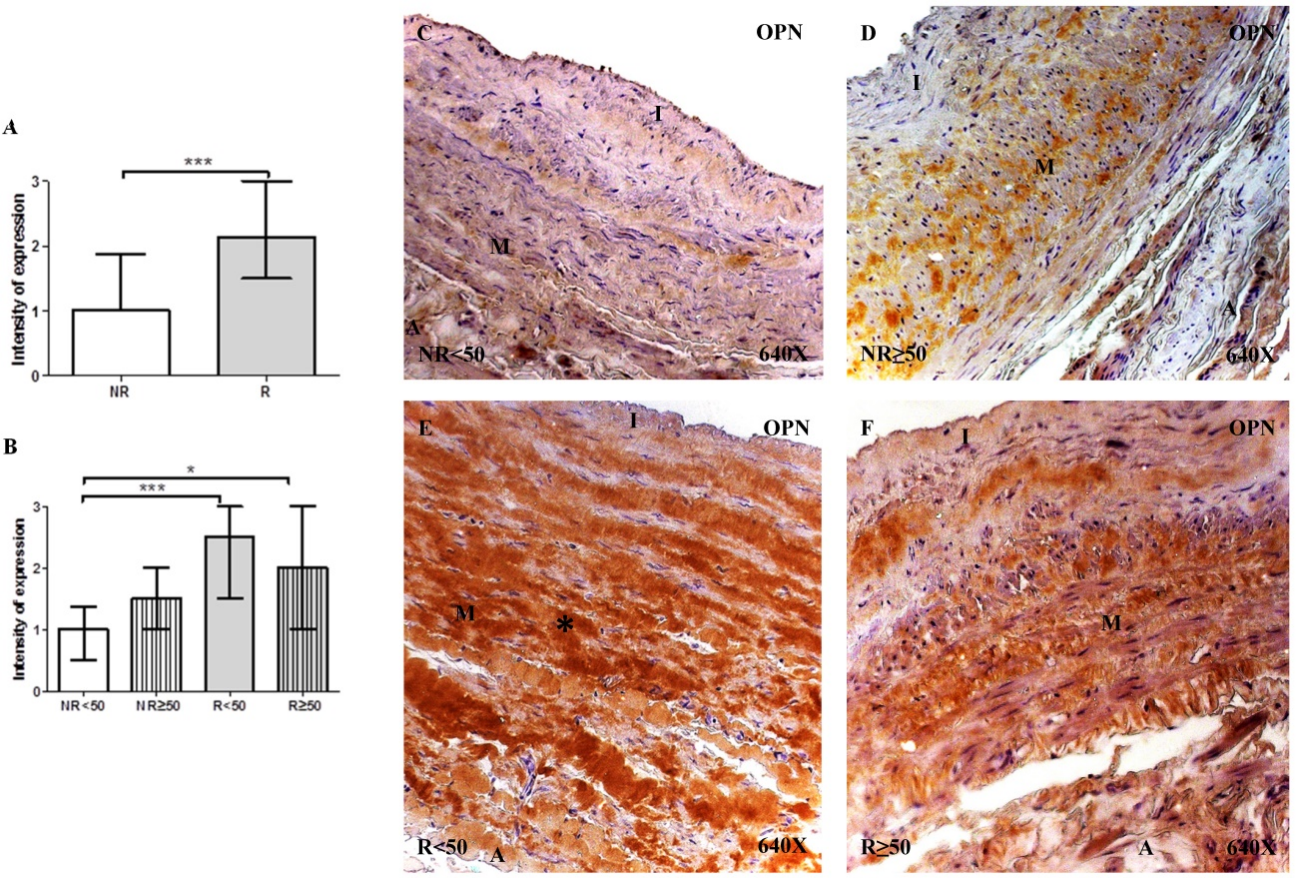
** A-B.** Intensity of Osteopontin (OPN) protein expression in venous wall in the different study groups. **C-F.** Images of protein expression by Osteopontin immunohistochemistry (OPN) by Immunohistochemistry technics in patients NR <50 (A), NR> 50 (B-C), R <50 (D-E) and R> 50 (F). Immunoprecipitation is brown. NR<50: patients without reflux younger than 50 years, NR≥50: patients without reflux with 50 years or older, R<50: patients with reflux younger than 50 years, R≥50: patients with reflux with 50 years or older. I, intimal layer; M, middle layer; A, adventitial layer. *p<0.05, ***p<0,001.

The determination of OPN protein expression using immunohistochemical techniques revealed that for patients with R, expression was observed in 100% (n = 81) of the samples, while for patients with NR, expression was observed in 89.65% (n = 26) of the samples. Regarding patient age, only patients with NR < 50 years old did not present total expression (76.92%, n = 10). The intensity of OPN expression was significantly higher in patients with R compared to the NR group (NR= 1.00 [0.00-3.00] vs R= 2.12 [0.50-3.00], ***p<0.0001). When considering the age factor, it is observed that there is a significant increase in patients R <50 compared to NR<50 (NR<50=1.00 [0.00-3.00] vs R<50= 2.50 [1.00-3.00], ***p = 0.0002) and in patients R≥ 50 compared to NR≥50 (R≥ 50=1.50 [0.75-3.00] vs NR≥50=2.00 [0.500-3.00], *p=0.0146). OPN protein expression was localized in the extracellular matrix of the 3 layers of the venous wall (Figure 4C-F). Notably, patients with R presented a greater OPN expression intensity, which was particularly noticeable in the middle layer of patients with R < 50 years old (Figure 4E, *asterisk). Fisher´s exact test showed a statistical relation between NR vs. R (p = 0.004).

### Higher expression of Pigment Epithelium-Derived Factor (PEDF) in CVI patients

**Figure 5:**
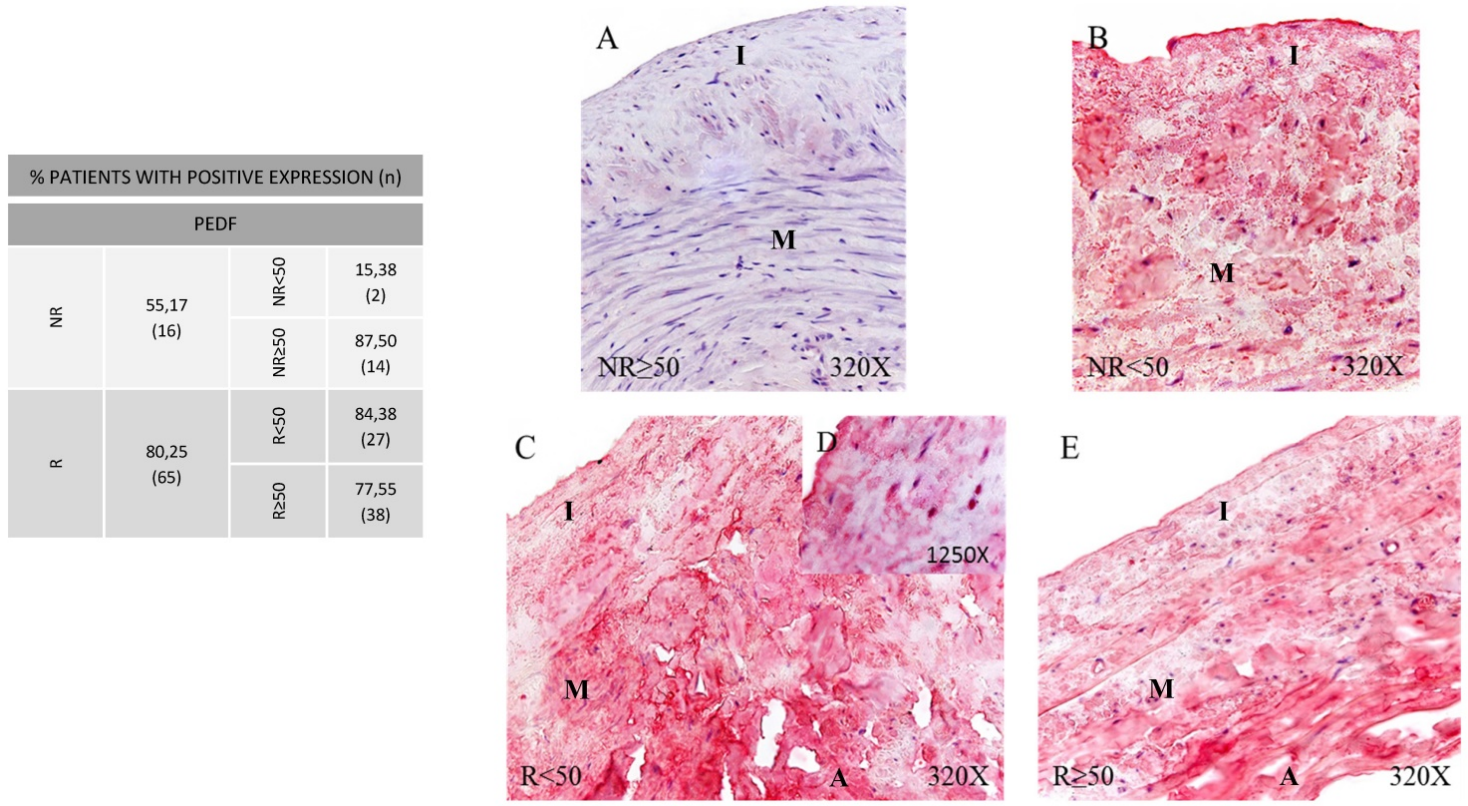
** Panel. A.** Percentage distributions of patients showing positive protein expression for Pigment Epithelium‐Derived Factor (PEDF) by immunohistochemistry technics in patients without venous reflux (NR) and with reflux (R) stratified by age, n=number of patients. **Panel. B.** Images show the protein expression of PEDF through specific immunohistochemical detection in the patient groups NR<50 (A), NR≥50 (B), R<50 (C, D) and R≥50 (E). The specific precipitate correlated with the expression of this protein in the different vein layers appears in red. NR<50: patients without reflux younger than 50 years, NR≥50: patients without reflux with 50 years or older, R<50: patients with reflux younger than 50 years, R≥50: patients with reflux with 50 years or older. I, intimal layer; M, middle layer; A, adventitial layer.

In PEDF protein expression analysis, once again greater expression was observed in the patients showing venous reflux (80.25%) compared to the NR group (55.17%) (Figure 5, Panel A). By age, patients NR≥50 (87.50%) and patients R<50 (84.38%) showed the higher PEDF protein expression percentages compared to the remaining patient groups. When detected, expression proportions were similar across the groups with a trend observed towards a greater labelling intensity in patients R<50 (Figure 5, Panel B. C-D). This process seems to acquire more relevance with age, and is also related to venous reflux as observed in our group of younger participants with valve incompetence. Fisher´s exact test showed a statistical relation between NR vs. R (p = 0.013).

### Presence of amorphous calcification and osseous metaplasia in venous wall of CVI patients

**Figure 6:**
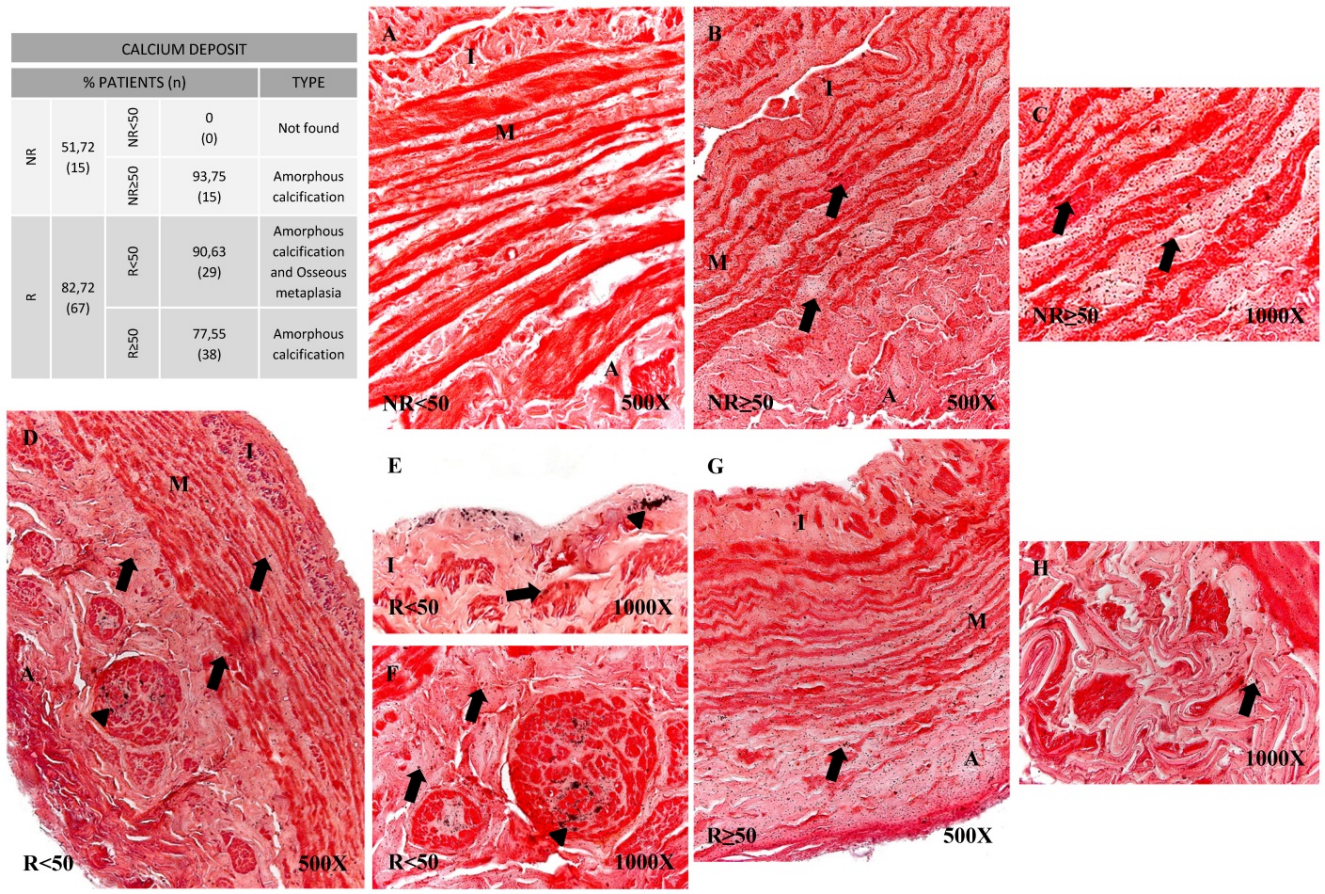
** Panel. A**. Percentage distributions of patients with calcium deposits and calcification types by Von Kossa technics in patients without venous reflex (NR) and with reflux (R) stratified by age, n=number of patients. **Panel. B.** Images show calcium deposition in the different vein layers in the patient groups NR<50 (A), NR≥50 (B-C), R<50 (D-F) and R≥50 (G-H). Black punctate deposits are amorphous calcification (arrow), while dense irregular deposits are osseous metaplasia (arrowhead). NR<50: patients without reflux younger than 50 years, NR≥50: patients without reflux with 50 years or older, R<50: patients with reflux younger than 50 years, R≥50: patients with reflux with 50 years or older. I, intimal layer; M, middle layer; A, adventitial layer.

Using histological techniques (Von Kossa) we were able to detect calcium deposits in the vein wall. These deposits were observed in greater proportions of patients showing venous reflux (51.72% NR vs 82.72% R). When age groups were compared, it emerged that in the younger patients with no reflux (NR<50), no calcifications were observed unlike in the remaining patient groups (Figure 6). Different calcification patterns were observed in the study participants. Most of the patients in NR≥50 (93.75%) and R≥50 (77.55%) were positive for amorphous calcification (Figure 6B-C and G-H). This pattern was described as isolated points of labelled calcium detected throughout the vein wall. In the younger patients with valve incompetence R<50 both osseous metaplasia and amorphous calcification were observed (90.63%) (Figure 6D-F), the former pattern being dense and irregular (Figure 6D-F, arrowhead). Furthermore, it is observed in the intimal layer (Figure 6E). Fisher's exact test showed a statistical relation (p = 0.001).

Using a tabletop TM electron microscope (TM-100), calcium levels were found to be significantly greater in the patients with venous reflux (***p = 0.0006). When age was taken into account, this significant difference was maintained in the younger individuals (*p = 0.05). In both the NR and R groups there was a trend towards greater calcium levels in the older subjects.

**Figure 7:**
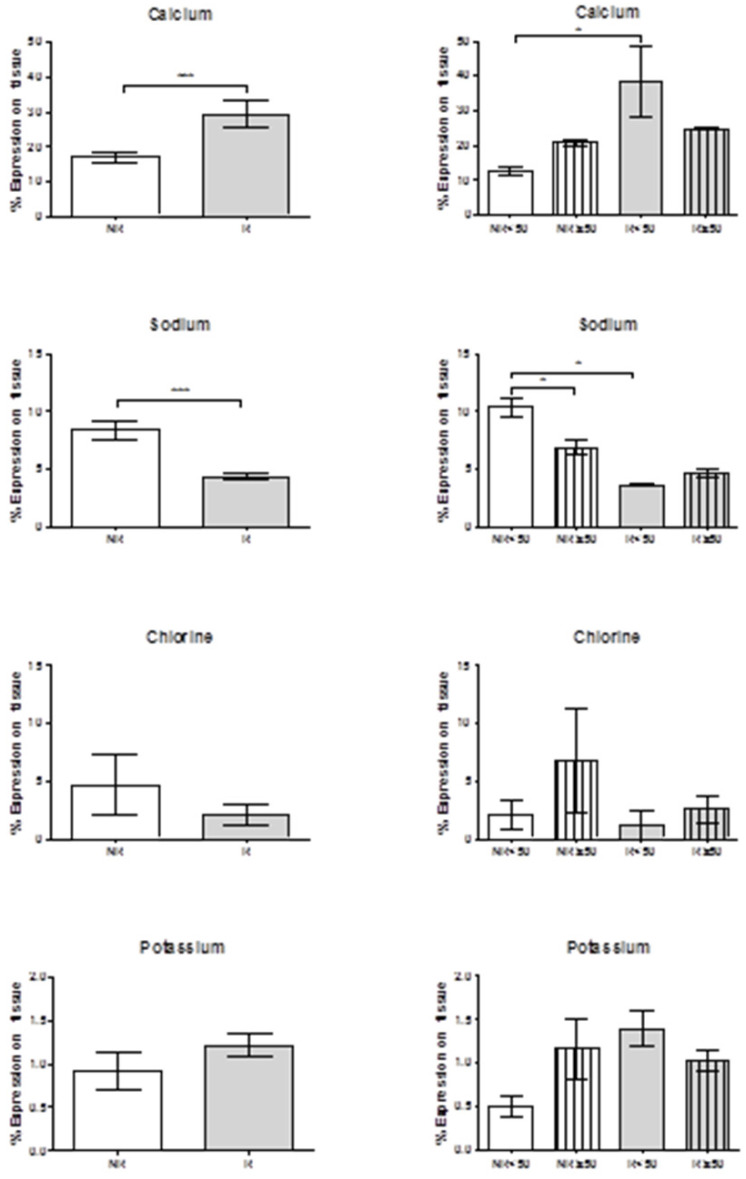
Histograms show expression percentages for calcium, sodium, chlorine and potassium by transmission electron microscopy (TEM) technics throughout the vein tissue in the different groups according to patient age. *p<0.05, **p<0,005. NR<50: patients without reflux younger than 50 years, NR≥50: patients without reflux with 50 years or older, R<50: patients with reflux younger than 50 years, R≥50: patients with reflux with 50 years or older.

Sodium levels were significantly reduced in the patients showing valve incompetence (***p = 0.0009). Further, in younger patients with no reflux (NR<50) sodium levels were significantly higher than in older patients showing NR (*p = 0.0317), and also higher than in the younger patients with venous reflux (*p = 0.05). Chlorine showed a tendency towards lower levels in the patients with reflux regardless of age. Potassium levels were inversely related to chlorine and tended to be higher in the patients with valve incompetence. We should highlight that patients NR≥50 and R<50 showed high potassium levels that were inversely proportional to levels of chlorine and sodium (Figure 7).

### Venous wall of patients with CVI has a greater presence of fibrinoid deposits

**Figure 8:**
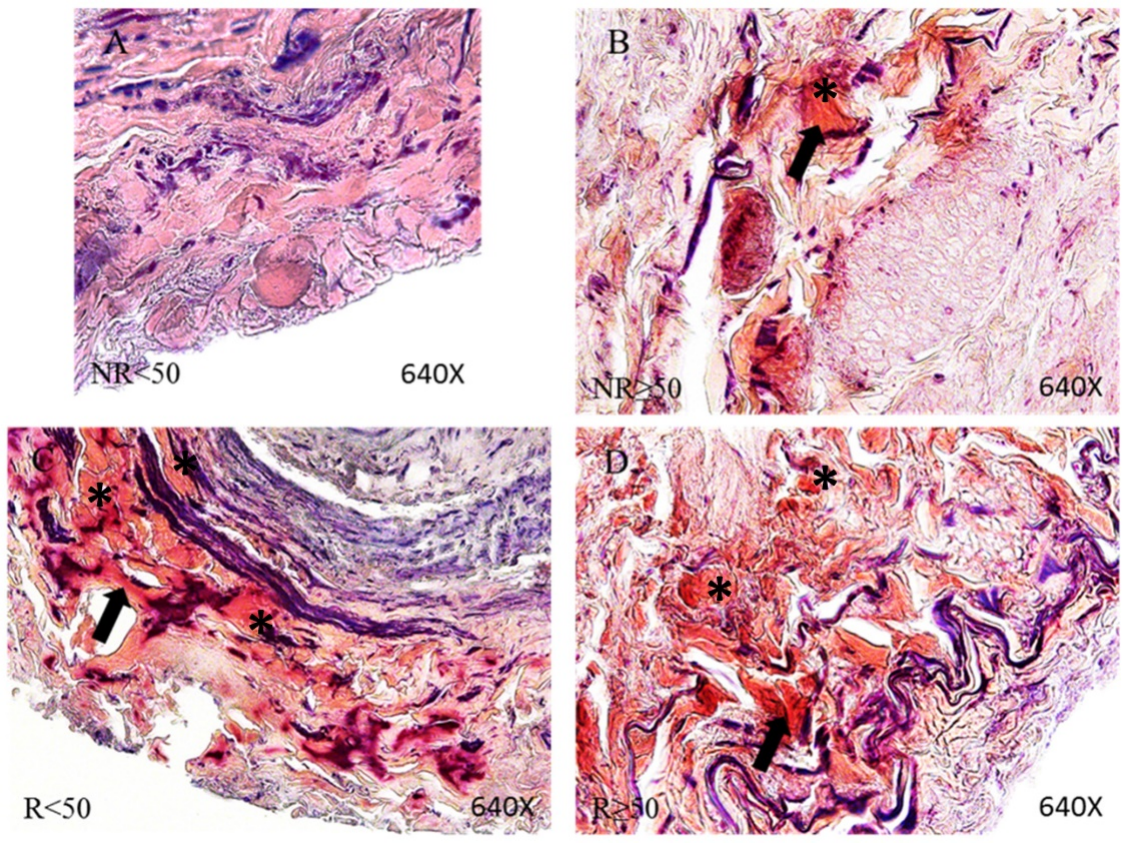
Images showing fibrinoid deposits stained by Phosphotungstic acid haematoxylin (PTAH) technics in the vein adventitious layer (arrow and asterisk) in the patient groups NR<50 (A), NR≥50 (B), R<50 (C) and R≥50 (D). NR<50: patients without reflux younger than 50 years, NR≥50: patients without reflux with 50 years or older, R<50: patients with reflux younger than 50 years, R≥50: patients with reflux with 50 years or older.

Through PTAH staining we were able to identify fibrinoid material in the vein walls of all our patient groups except the NR<50 group in which only 3 patients showed these deposits. Deposits observed in 100% of the patients in the other groups appeared in the adventitious layer of the vein and at the boundary between this layer and the middle layer. In Figure 8, fibrinoid deposits can be noted in the reddish zone in the adventitial layer of the vein wall (arrow).

## Discussion

Valve incompetence, clinically diagnosed as venous reflux, leads to venous hypertension, which triggers a change in tissue homeostasis 49,50. In this sense, our results show how venous reflux is correlated with an increase in the protein expression of JNK and of bone metabolism-related markers. Additionally, a significant increase in the expression of JNK, RUNX2, OSC and OSP was observed in younger patients with venous reflux. These findings suggest that younger patients with venous reflux undergo cellular activation processes and metabolic changes, perhaps to satisfy the demand caused by the pathology. Our study shows the presence of osseous metaplasia in these patients, which may indicate that the venous wall undergoes a distension that severely damages the different layers, which has systemic repercussions.

Vascular diseases are related to changes in JNK expression 22. Hui 51 showed that JNK plays a role in arteriosclerotic lesions. A histopathological analysis by Metzler et al. 52 demonstrated elevated JNK expression in smooth muscle cells in vascular disease. Our results show, in agreement with prior studies, that the smooth muscle cells of patients with CVI have increased JNK expression. Wu et al. 28 demonstrated *in vitro* that JNK expression was directly related to the presence of calcifications in smooth muscle cells of the vascular system. One of the relevant findings of our study is the co-localization of JNK and RUNX2 protein expression, as both were localized in the smooth muscle cells of the middle layer of these patients and were significantly higher in patients with venous reflux. Recent studies have pointed to the relationship between the 2 and its importance in osteogenesis 53,54. Papachristou et al. 55 noted the importance of the relationship between the JNK and RUNX2 pathways at the histopathological level. Experimental models have shown that JNK and RUNX2 are involved in the calcification of smooth muscle cells of the vascular system in disease situations 56,58. This supports the results of our study regarding the existing relationship between the 2 molecules and their involvement in vascular pathology. One of the limitations of our study is to know the expression downstream, as c-Jun and other as p-JNK. Future studies are necessary in this way.

Vascular calcification often occurs with advanced age or in genetic diseases, leading to serious clinical consequences. Patients with chronic venous disease show increased activity of genes related to altered matrix mineralization, such as matrix Gla protein (MGP) 58. These authors showed that the inhibition of the expression of this protein improves proliferation and mineralization processes through impaired MGP carboxylation.

Studies indicate the existence of a profile of differentially expressed genes in varicose veins, in particular components related to extracellular matrix impairment 2,9,19,59,60. In this sense, calcification of varicose vein walls is a consequence of the pathology and the changes associated with it. This mineralization can occur throughout the entire length of the venous wall, involving different types of calcification. Kawakami et al. 33 noted that vascular system calcification is the result of numerous causes that trigger inflammatory and osteogenic processes or disorganized mineral homeostasis. Recent studies have shown how patients have shown an increase in calcification in relation to aging, chronic kidney disease and serum creatinine in relation to endothelial damage 60. This study determined how in these patients venous calcification could show a pattern similar to arterial calcification with changes in the extracellular matrix. Our studies are in relation to what was described by these authors, also showing that calcification of the extracellular matrix may be related to the acceleration of osteogenic differentiation of vascular cells and their relationship with an aging process. Ortega et al. 19 showed that in venous reflux, younger patients in particular had greater activation of proinflammatory cells, which is consistent with what has been observed in other studies in the arterial system as a trigger for the onset and alteration of osteogenic processes.

Numerous studies have demonstrated that osteogenesis is present in arterial disease, but it is not yet known if this is true for the venous system of the lower limbs. Currently, the study of vascular biology considers osteogenesis a fundamental process for understanding tissue failure 37,61-63.

RUNX2 has been shown to play a determinant role as a promoter of vascular system calcification because it serves as a marker of the transdifferentiation of vascular smooth muscle cells into an osteogenic phenotype 64-66. Byon et al. 66 showed that RUNX2 inhibition had consequences on tissue calcification. Our results demonstrated that the expression of RUNX2 was significantly higher in patients with valve incompetence (venous reflux) than in controls and that younger patients had the highest expression levels.

The patients included in the present study suffer from a chronic disease; therefore, when an acute response is not resolved and becomes chronic, the same proteins that once promoted healing contribute to chronic inflammatory pathologies, such as calcification. Some of the matricellular proteins that we observed to be increased were OCN and OPN. These proteins are highly regulated in acute and chronic inflammatory environments and have been implicated in physiological and pathophysiological processes 67-71. Zhang et al. 72 showed increased OCN and RUNX2 activity in the calcification of smooth muscle cells. Similar results were shown by Miller et al. 73 in an *in vitro* study. These findings support the results found in patients with valve incompetence (venous reflux). In this sense, studies of the genetic expression of the proteins involved must be carried out, as well as knowing possible related mutations. This being one of the limitations of our studies. However, our study manages to approximate at a tissue level a fact of great importance in the knowledge of venous pathology.

Another important and controversial point in the current literature is the role of OPN in cardiovascular disease. Some authors suggest that it as an attenuator of vascular calcification in acute processes, in contrast with other studies that show its osteogenic role in chronic processes 74,75. To understand the double role that OPN may play in venous disease, its demonstrated role in neovascularization processes in response to ischaemia must be considered 76. Previous studies demonstrated an increase in hypoxia-inducible factors in patients with venous reflux 19. This could explain the possible role of OPN in neovascularization in patients with chronic venous disease. Neovascularization is a known and clinically verified finding in patients with venous disease 77. However, the main role attributed to OPN is its involvement in the inflammation produced in peripheral vascular disease 78, 79. It has been shown that OPN levels were elevated in patients with asymptomatic calcified aortic valve disease 80. In contrast, a study with OPN-/- mice showed that OPN played an inhibitory role in vascular calcification 81. Sainger et al. 82 noted that the key to understanding this duality is the phosphorylation of the molecule. Yadav et al. 83 demonstrated that the phosphorylation of OPN becomes impaired with age.

Another factor related to vascular diseases, which our results show, is the expression of PEDF. The activation of PEDF has been mentioned in relation to extracellular matrix mineralization 39, 84. Therefore, these molecules may play a central role regulating the development of vascular calcification that coincides with decreased skeletal mineralization with age, osteoporosis or disease 85. Boraldi et al. 86 showed that PEDF was related to the calcification of elastic fibres. In this sense, PEDF may have a relevant role in the calcification of the venous system in CVI. Shiga et al. 87 showed that serum PEDF levels were altered in patients with chronic vascular diseases. In this sense, Demer & Tintut 88 described that the increase in molecules related to oxidative stress and lipid peroxidation is related with vascular calcification. This state can produce tissue changes that could be related to findings such as the fibrinoid deposit. It is known that fibrinoid deposition acts in tissue and cellular alterations, characterized by the remodeling of tissue proteins. Recent studies have shown that *in situ*ations of vascular disorders, highly vascularized tissues and blood vessels have fibrinoid deposits 89, 90. All of these findings suggest that these patients suffer chronic damage with a cellular response that manifests as elevated activation of the JNK pathway, which can occur not only locally at the tissue level but also at the systemic level. In this sense, future studies should evaluate the role of proinflammatory molecules and extracellular matrix remodeling in correlation to the expression of these molecules with markers of bone metabolism and JNK.

## Conclusions

We conclude that venous reflux causes an increase in the activation of cellular signaling of PEDF which contributes to mineralization of extracellular matrix (amorphous calcification). This calcification activated the JNK pathway, triggering osteogenic differentiation of vascular cells mediated by different mechanisms that increased the osseous metaplasia in venous wall, such as those described in our study. This suggests that our results are associated to ageing in patients with venous reflux, as younger patients showed increased JNK, RUNX2, OSC, OSP and PEDF expression. However, our study has limitations such as semi-quantitative method for detecting proteins expressed in the tissues, therefore presents the limitation of an observational study and is necessary *in vitro* studies. Despite this, our study presents a novelty of great importance in the study of venous disease. This increased expression may be considered a finding of premature and accelerated asynchronous ageing in these patients, possibly in subjects with a certain genetic background. Therefore, in younger patients with CVI, expression of the JNK pathway becomes an important and determining factor with consequences on the venous system.

## Figures and Tables

**Table 1:**
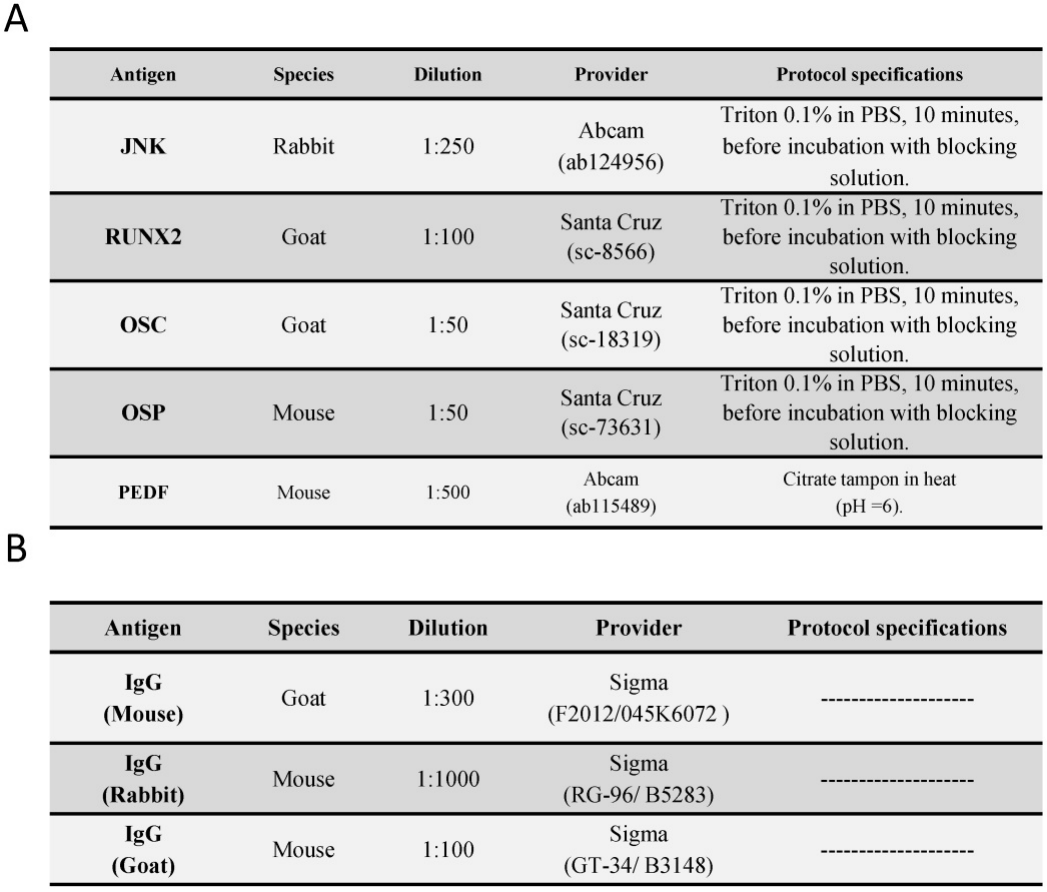
Primary (A) and secondary (A) antibodies used in the immunohistochemical studies performed, showing the dilutions used and the specificities in their protocol
